# Incubation Patterns in a Central-Place Forager Affect Lifetime Reproductive Success: Scaling of Patterns from a Foraging Bout to a Lifetime

**DOI:** 10.1371/journal.pone.0017760

**Published:** 2011-03-15

**Authors:** Akiko Shoji, Kyle H. Elliott, Stéphane Aris-Brosou, Doug Crump, Anthony J. Gaston

**Affiliations:** 1 Department of Biology, University of Ottawa, Ottawa, Ontario, Canada; 2 National Wildlife Research Centre, Environment Canada, Ottawa, Ontario, Canada; 3 Department of Zoology, University of Manitoba, Winnipeg, Manitoba, Canada; Institute of Marine Research, Norway

## Abstract

**Background:**

Long-lived seabirds face a conflict between current and lifelong reproductive success. During incubation shifts, egg neglect is sometimes necessary to avoid starvation, but may compromise the current reproductive attempt. However, factors underlying this decision process are poorly understood. We focus on the ancient murrelet, *Synthliboramphus antiquus*, an alcid with exceptionally long incubation shift lengths, and test the impact of environmental factors on incubation shift length in relation to reproductive success.

**Methodology/Principal Findings:**

Using an information theoretic approach, we show that incubation shift length was a strong predictor of reproductive success for ancient murrelets at Reef Island, Haida Gwaii, British Columbia, Canada during the 2007 and 2008 breeding seasons. The most important factors explaining an individual's shift length were egg size, wind speed and the length of the mate's previous shift. Wind speed and tide height were the two most important factors for determining foraging behavior, as measured by dive frequency and depth.

**Conclusions/Significance:**

Our study demonstrates that (i) species-specific reproductive strategies interact with environmental conditions such as wind speed to form multiple incubation patterns and (ii) maintaining regular incubation shifts is an essential component of reproductive success.

## Introduction

Life-history theory predicts that fitness is optimized by balancing investment in current reproduction with costs of reducing an individual's ability to invest in future reproduction [1]. One set of life-history decisions that birds face during the breeding season is the timing, frequency, and duration of their visits to the nest [2]. These decisions are reflected by the activities of individual birds, who try to maintain body condition and minimize predation risk [3], with the ultimate goal of maximizing reproductive fitness.

Most seabirds have a biparental incubation strategy and therefore alternate fasting bouts with foraging trips to replenish their body reserves [4], [5], [6]. Seabirds undergo large variations in body mass during their breeding period [7], [8], [9] and the rate of breeding failure can be high during the incubation period [10]. Maintaining incubation shift length in seabirds is critical and this is especially true for ancient murrelets *Synthliboramphus antiquus* which do not feed their chicks at the nest. Therefore, unlike nearly all other seabirds, incubation in this species is expected to be the most demanding phase of breeding. Nevertheless, most studies have focused on behaviour during the nestling period [11], [12], [13], [14], [15], [16], while incubation shifts have received less attention.

Interruptions to incubation occur routinely in many species of birds [17]. Such interruptions, when normal incubation behavior is resumed, are known as “egg neglect”. Temporary egg neglect may be observed during successful breeding (*e.g*. northern fulmar *Fulmarus glacialis* [18], blue petrels *Halobaena caerulea* [19], Cassin's auklet *Ptychoramphus aleuticus* [16]), but egg neglect is also known to increase the probability of breeding failure [19]. The ultimate manifestation of egg neglect, desertion (incubation is terminated permanently), is itself presumed to be the consequence of life-history costs and benefits [20]. One of the determinants of nest desertion is the length of foraging trips [15], as there may be a physiological limit beyond which an incubating bird will abandon the nest when its partner has been out foraging for “too long” [19], [21], [22], [23]. Therefore, it is important for a particular bird to decide on how long it can remain out at sea foraging so that its partner does not leave the nest. This reasoning suggests that incubation shift length should be synchronized between pair members to match to their partners' ability to fast [24], [25].

It has been suggested that both colony attendance and diving behavior are influenced by unusually poor foraging conditions such as high wind speed [3] and small tidal currents [26], and that egg neglect may be an indirect consequence of these factors. In seabirds, trip duration may be prolonged due to unpredictable weather conditions and patchy prey distribution [27], [28] (but see [12]). Indeed, longer trips may allow for longer search times and for more encounters with rarer but energetically more valuable prey items or patches [29], [30], [31], [32], which allow a particular foraging bird to have a longer shift. As a result, the incubating partner has to fast for an extended period of time, which depletes its body reserves [19], [33], and can lead to egg neglect. On the other hand, when feeding conditions are good, birds are expected to spend less time away from their nest. We will refer to the idea that incubation shift duration is determined primarily by foraging conditions as the “foraging hypothesis”.

However, incubation behavior may also be influenced by predation risk [16], [34], [35]. While many seabirds are diurnal, the majority of petrels (e.g. Procellariidae, Hydrobatidae) and some auks (Alcidae) that are vulnerable to predation on land, are nocturnal on their breeding grounds. Previous studies have suggested that nocturnal colony attendance among these seabirds is an adaptation to minimize predation risk from diurnal avian predators [36], [37], [38]. Nocturnal seabirds reduce nighttime activity and alter the timing of their visit to the colony on moonlit nights, supporting the idea that nocturnal visitation reduces predation risk [3], [39]. Despite nocturnal colony attendance, ancient murrelets are frequently killed by predators on their breeding grounds, presumably while returning to or departing from their nests [40]. Reducing the frequency of colony visits by extending incubation shift lengths in nocturnal, burrow-nesting marine birds is therefore expected to be an adaptive strategy further reducing their mortality from predators. Under this hypothesis parents may adjust the number of visits during the nesting season in order to maximize their life-time fitness [40]. We will refer to this hypothesis explaining incubation shift duration as the “predation hypothesis”.

In this study, we test these two non-mutually exclusive hypotheses, foraging and predation, as determinants of incubation behavior, with a particular emphasis on the duration of shifts, in the ancient murrelet. During incubation, adults nest in underground burrows and visit the breeding colony only at night. Members of this genus frequently neglect their eggs for a few days, and their mean incubation shifts are long (mean  = 3 days, range  = 1–6 days, [40]) compared to other members of the auk family. Yet, the reason for this difference in incubation shift length is unclear [40], [41], [42], [43]. Here, we first assess the extent to which environmental factors explain variation in shift lengths observed in murrelets, by testing whether longer foraging bouts increase the probability of egg neglect by their partner and whether this result affects reproductive success. Based on available information from previous studies, we predict that incubation shift length, therefore also egg neglect, should increase with: (i) increasing wind speed [44], and (ii) increasing nighttime light intensity [45]. We also predict that shift length should be affected by synchronization of incubation shift length between partners [46], which in turn should affect reproductive success. We show that our predictions hold true in ancient murrelets at our particular study site, which provides us with a novel understanding of the proximate factors influencing the incubation shift length in a seabird and of the importance of environmental conditions on reproductive success.

## Materials and Methods

### Ethics Statement

All field procedures were approved by the Animal Care Committee of the National Wildlife Research Centre and the Ontario Region of Environment Canada operating under the guidelines of the Canadian Committee for Animal Care (Permit Number 0700AG02, 0800AG02, 0900AG02).

### Field methods

The study was conducted on Reef Island (52°52′N, 131°31′W), Haida Gwaii, British Columbia, Canada. The experimental part of the study was conducted during April 5^th^ to June 29^th^ 2007, and April 4^th^ to June 12^th^ 2008. Ten days of additional fieldwork were carried out in May 2009 to obtain further data on diving behavior. The Reef Island colony supports approximately 5,000 pairs of ancient murrelets [47]. General monitoring procedures for nest attendance patterns in murrelets were followed [17]. The majority of burrows are in soft soil susceptible to damage by investigators. Consequently, artificial nest boxes were used to monitor incubation behavior (*n* = 72; see [48] for details). These boxes were installed ten years prior to the study, in 1997. The total number of occupied boxes (at least one egg laid) was 58 in 2007 and 61 in 2008. Normal clutch size in this species is two and eggs were marked to identify laying order. Their length and width was measured to calculate the egg volume index (Length × Width^2^) as a proxy for egg volume.

We set up knock-down tags, made of small twigs placed at the entrance of burrow nests. This technique is widely used to monitor seabirds' activity in and out of their burrow. Nest boxes were inspected only when tags were displaced [49]. In spite of their wide use in studies of burrow nesting seabirds [40], knock-down tags do not allow us to distinguish individuals, or to tell which sex is coming in or out of the borrow. However, knock-down tags provide a reliable index of nest attendance patterns, with an accuracy estimated to be 79% (range: 61–96%) [43], [49]. Once the first egg was laid, either a temperature sensor (Onset TMC1HD) or a YSI temperature probe (Yellow Springs Instruments; 400 series) was inserted into the nest chamber so that progress of incubation could be monitored (i.e. presence or absence of incubating birds). The temperature sensors were connected to an electronic recorder (Onset H08-006-04) and downloaded daily. The YSI temperature probe was connected to a telethermometer and read once daily, as ancient murrelets only enter or exit their burrows at night [40]. We secured the tips of the temperature probes in the nest cup with thumbtacks fixed onto the floor of the box during the daytime, prior to the laying of the second egg, when birds were not present. In 2008, we attached 17 transmitters randomly to one partner of each of 17 pairs. Nest attendance patterns were checked once daily during the daytime. At each check, shift changes were scored as either “change” or “no change”. The knock-down method indicates “change” when tags at the entrance have been displaced. The radio telemetry method indicates “change” when radio signals have appeared or disappeared (because only one member of each pair was fitted with a transmitter). Otherwise the shift status was scored as “no change”.

In 2007, vocalization activities were recorded by inserting a microphone attached to an mp3 recorder into occupied nest boxes (*n* = 10). Recorders were operated from 10:00 pm to 05:00 am, the period when changeovers may occur (time was recorded as PDT - Pacific Daylight Time). An immediate burst of chatter-calling by both members of the pair signaled the arrival of the foraging or ‘off-duty’ bird; we used this signal to determine when the off-duty bird returned to the nest.

After 30 days of incubation, at least one bird per nest was weighed and 1 ml of blood was sampled and stored on protein-saver paper for genetic sex determination using a chelex DNA extraction technique [50] and the P2/P8 Polymerase Chain Reaction method [51]. In cases where only one pair member was sexed, the other pair member was assumed to be of the opposite sex. Handling times were kept <3 min.

To measure the number, duration and depths of dives during foraging trips, incubating birds were captured during daytime in their nest box and equipped with cylindrical time depth recorders (TDRs). Between April 27^th^ and May 20^th^ 2008, two birds were fitted with TDRs (Lotek 1100 LTD; sampling interval  = 3 s; memory  = 128 Kb [55 hours]; weight  = 5 g; diameter  = 1 cm; length  = 3.3 cm; accuracy  =  ±2 m; [52]). Between April 28^th^ and May 12^th^ 2009, four birds were equipped with the same Lotek 1100 LTD TDRs and eight with the lighter and more accurate Lotek 1500 LAT (sampling interval  = 4 s with 1 s sampling when below 2 m for murrelets; memory  = 512 Kb; weight  = 3.2 g; diameter  = 0.5 cm; length  = 3.3 cm; precision  =  ±0.25 m). We used adhesive tape to attach each device to the tarsus, without any additional metal band. Due to device measurement uncertainty, only depths >2 m were considered to be actual dives. To minimize any bias associated with the daily light cycle and because there were virtually no dives during nighttime, all dives between 22:00 to 04:00 were excluded, as murrelets are diurnal feeders [53], [54].

### Weather conditions and ambient light

**Figure 1:**
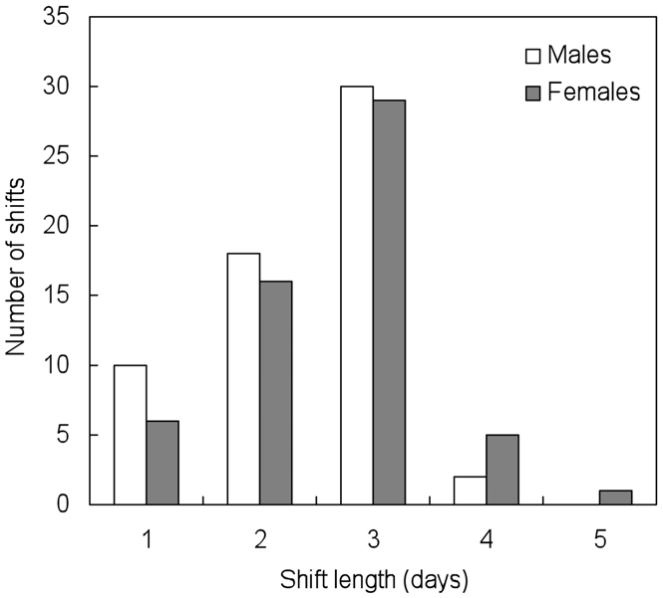
Distribution of incubation shift lengths of ancient murrelets at Reef Island measured by radio-telemetry at the artificial nest boxes in the study plot in 2008 (male: *n* = 61, female: *n* = 58).

Hourly wind data were obtained from the closest weather station (Sandspit, 40 km to the north, www.weatheroffice.gc.ca). A previous study showed that high wind speeds decrease the ability of ancient murrelets at Reef Island to forage at sea at various time scales [3]. Thus, we used mean wind speeds over two different time scales (6 h and 72 h prior to a particular night) to assess the effect of foraging conditions on incubation shift length. The 6 h period preceding arrival at the colony corresponds to the bird's return journey from the foraging area, as birds begin to arrive at the colony several hours before dusk [40]; 72 h durations correspond to the length of typical foraging trips (modal incubation shift length at present study site 2–3 days, Fig. 1).

Hourly wave height data were obtained from the North Hecate Strait buoy station (www.dfo-mpo.gc.ca), which is the closest station recording such data, about 91 km away NE from our study site (www.movable-type.co.uk/scripts/latlong.html). Tide height was obtained from the Queen Charlotte City station (www.waterlevels.gc.ca). We further obtained lunar phase (or moon age; www.timeanddate.com) and daily cloud opacity (measured in tenths of the outdoor light; www.weatheroffice.gc.ca).

### Effects of partner's behavior

We used the knock-down method described above to monitor incubation shift length of each partner: we considered a changeover to have occurred when tags were knocked down [49]. Comparisons of incubation shift lengths within pair members within a given year were made using the Spearman correlation coefficient as incubation shift length departed from normality.

### Reproductive success

After 30 days of incubation, the reproductive success of each pair was checked [55]. Forty-one of 58 and 35 of 61 pairs produced at least one chick to departure in 2007 and 2008, respectively. Eggs depredated by deer mice (the only mammalian predator present on the island) were easily distinguished from their hatched counterparts by tooth marks on shell remains [56], [57].

### Data analysis

We performed all analysis with R 2.6.1 [58]. In this study, we used either generalized linear models (GLZ) to model adult nest arrival and egg neglect, or generalized linear mixed model (GLMM) to model incubation shift length, reproductive success, dive depth and dive frequency. Model fit was checked with diagnostic scatter plots, using standard residuals, and transformations were applied where necessary.

The model-building procedure employed a multimodel inference approach [59], [60] to address hypotheses about temporal and weather-related patterns in shift length. [60], [61]. We considered all of the models that were biologically reasonable, which involved various combinations of the different independent variables. In all cases detailed below, models were ranked with AIC and ΔAIC was used to infer support for models in the candidate set [60], [61].

We modeled the proportion of parents returning to their nests and the proportion of eggs neglected (*i.e.* left unincubated for a period of at least 24 h) as a function of weather conditions. As these are proportion data, we used a Binomial distribution with a logit link function.

For incubation shift length, we assumed that egg size (Egg Volume Index) had an overarching effect on shift length because egg size is often related to female age and quality [42], [62]. Besides, if energetic limitation is important, we expect that the length of the previous shift would influence shift length. We therefore included this second variable in the models. We then tested whether timing of hatch influenced shift lengths by adding Julian date of hatch to the model. We controlled for site effects by considering nest identifiers (ID) as random effects. To model the effect that weather might play in day-to-day adjustment of incubation shift length, we added measurements of wind speed at two different times (6 and 72 h before nest arrival) and of visibility (moon or cloud). Finally, we tested the hypothesis that variation in hatch date and egg size modifies the relationship between previous shift length and shift length by including the corresponding interaction terms in our models. In total, we considered 10 candidate models for incubation shift length.

The models for reproductive success were specified to test the following hypotheses. First, we assumed that egg size influences reproductive success (see previous paragraph). Mean shift length was added to the models to test the effect of schedule coordination between pair members and year effect was added to test the effect of intra seasonal variation in reproductive success. Nest-specific correlation coefficients of incubation shift length between pair members (synchrony) and cumulative days of egg neglect were added to test those effects on reproductive success. Location effect was controlled by modeling nest ID as a random effect. Finally, we tested the hypothesis that variation in egg size and year modifies the relationship between mean shift length and nest specific correlation coefficients, and reproductive success by including the corresponding interaction terms in our models. In total, we considered 11 candidate models for reproductive success.

To test if dive depth frequency was related to weather conditions, we used similar GLZ modeling with Poisson distribution and a log link function. We considered wind speed and direction (circularly transformed), wave height, tide change (current tide height minus tide height one hour prior), tidal amplitude (difference between high and low tide) and time of day (circularly transformed) as independent variables. Individual identity was controlled by modeling this factor as a random effect. As weather might affect hour-to-hour adjustment of diving behavior at sea, we considered hourly weather data. Unless otherwise indicated, the results are expressed as means ± 1 SD and all reported *P*-values are two-tailed.

## Results

### Timing of nest arrivals

The exact timing of returns to the colony, as documented by recordings of vocalizations, was only available for 2007. In this year, the average time of nest arrivals was earlier during the incubation period (23:29, *n* = 136, mean incubation period: 10 May; range: 20 April-31 May) than during the pre-incubation period (00:17, *n = *47; mean pre-incubation period: 19 April; 12 April-1 May, *t* = 6.89, *P*<0.0001). During the incubation period, the first nest arrivals on each night occurred about two hours after nautical twilight, and as the time of sunset changed throughout the season, the timing of first arrival was slightly but significantly correlated with calendar date (*r* ^2^ = 0.15, *P*<0.0001). In contrast, timing of arrival was not significantly correlated with the calendar date during the pre-incubation period (*r* ^2^ = 0.07, *P* = 0.08). This result suggests that the time of nest arrival during the incubation period was regulated by light conditions, but that this was less so during the pre-incubation period. Based on sound recording data, there was no significant difference in nest arrival time among individual nests (Kruskal*-*Wallis test: *KW* = 13.4, *P* = 0.20).

### Adult nest arrival

The best model predicting the proportion of adults returning to their nests on a given night included wind speed and visibility (Table 1). Arrival rate was inversely related to wind speed during the 72 h prior to nest arrival, which suggests that foraging conditions at sea have a strong effect on the timing of return to the nest. Likewise, wind speed during the 6 h prior to nest arrival decreased adult nest arrival, which may interfere with navigation while returning to the colony. A similar effect was found for increasing lunar phase, while cloud opacity had no effect on arrival rate. These results suggest that more birds arrived at their nests when the ambient light intensity was low, which is consistent with the predation hypothesis.

**Table 1:** Results of AIC model comparison to explain adult nest arrivals and egg neglect of ancient murrelets.

Variables	Model	AIC	ΔAIC	*Wi*	*K*	Deviance
Arrival	X72H+mphase+X6H	2809.0	0.0	0.50	4	2369.0
(*n* = 98)	X72H + mphase + X6H + copacity	2809.0	0.0	0.50	4	2366.6
	X72H + mphase	2861.0	52.0	0.00	3	2422.6
	X72H	2983.0	174.0	0.00	2	2547.4
	Null	3128.0	319.0	0.00	1	2694.3
Neglect	X72H + mphase + X6H + copacity	1166.0	0.0	1.00	3	906.1
(*n* = 98)	X72H +mphase+X6H	1224.0	58.0	0.00	4	966.1
	X72H	1253.2	87.2	0.00	2	999.4
	X72H + mphase	1231.0	65.0	0.00	3	975.5
	Null	1277.4	111.4	0.00	1	1025.6

Notes- *Wi = * Akaike weight; *K* =  number of parameters; Arrival  =  percentage of parents returning to their nests in a given date; Neglect  =  percentage of egg neglected in a given date; X6H  =  wind speed 6 hours prior to colony arrival at 23:00; X72H  =  wind speed 72 hours prior to colony arrival at 23:00; mphase  =  proportion of moon (full moon: 14-day, new moon: 1-day); copacity  =  daily cloud opacity.

### Egg neglect

The model receiving greatest support suggested that the proportion of eggs neglected on a given night was related to the full model with wind speed, moon phase and cloud opacity (Table 1). Egg neglect increased with wind speed during the previous 6 h and 72 h prior to neglect. Cloud opacity decreased the proportion of egg neglect while ambient light conditions were positively correlated with the proportion of egg neglect. These results suggest that egg neglect is likely to be affected by both foraging conditions and risk of adult predation.

### Incubation shift length for known sex pairs

Of the 13 pairs for which one partner was equipped with a radio transmitter in 2008, incubation shift length did not differ significantly between males and females (female: 2.60±0.88 days, *n* = 58; male: 2.38±0.82 days, *n* = 61; *t* = −1.45, *P* = 0.15, Fig. 1). The mean duration of 119 shifts measured by radio telemetry was 2.49 days, and 56% lasted three days or more.

### Mate effects on shift length

**Figure 2:**
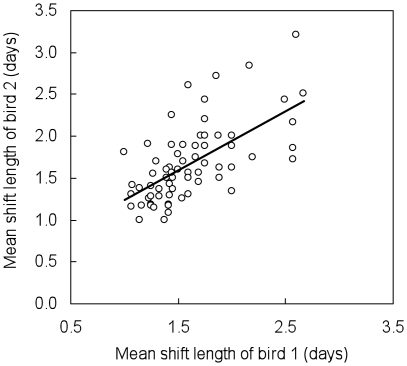
Incubation shift length was correlated between pair members in ancient murrelets (*n* = 75).

As predicted, incubation shift length was positively correlated with the duration of the previous shift by the partner (*r* = 0.29, *P*<0.05, *n* = 1385). In addition, the mean incubation shift length throughout incubation was positively correlated with that of the partner (*r* = 0.64, *P*<0.0001, *n* = 75, Fig. 2). These results were robust to the experimental technique used (knock-down tags), as telemetry also suggested a correlation of incubation shift length between alternating partners at a given nest (*r* = 0.33, *P*<0.05, *n* = 13). Mean incubation shift length differed among nests (*KW* = 259, *P*<0.0001).

### Factors affecting incubation shift lengths

The multimodel inference approach used here suggested that incubation shift length was influenced by the egg volume index, partner's previous shift, timing of breeding, and wind speed. We also found support for interactions among variables in these best main-effects models (Table 2). Removing short-term wind speed (6 h) increased AIC by >13 units, while removal of long-term wind speed (72 h) increased AIC by 5 units. Removal of the partner's previous shift and timing of breeding led to an increase of >26 units (see Table 2), suggesting that those variables strongly affect incubation shift length.

**Table 2:** Results of AIC model comparison to explain incubation shift length and reproductive success of murrelets.

Variables	Model	AIC	ΔAIC	*Wi*	*K*	Deviance
ISL	egg+pr_isl+end+X72H+X6H+egg*pre-isl	615.1	0.0	0.59	7	599.1
(*n* = 1305)	egg+pr_isl+end+X72H+X6H	615.8	0.7	0.26	6	601.8
	egg+pr_isl+end+X72H	628.2	13.1	0.00	5	616.2
	egg+pr_isl+end	633.0	17.9	0.00	4	623.0
	egg+pr_isl+end+moon	633.4	18.3	0.00	5	621.4
	egg+pr_isl+end+cloud	634.9	19.8	0.00	5	622.9
	egg+pr_isl	635.2	20.1	0.00	3	627.2
	egg	661.6	46.5	0.00	2	655.6
	egg+pr_isl+end+X72H+X6H+end*pre-isl	681.1	66.0	0.00	7	667.1
	Null	733.4	118.3	0.00	1	729.4
RS	egg +COR+neg+egg*ISL	84.3	0.0	0.36	5	70.3
(*n* = 76)	egg +COR+neg+year*ISL	84.4	0.1	0.34	5	68.4
	egg +COR+neg	84.7	0.4	0.30	4	74.7
	egg +COR	94.3	10.0	0.00	3	86.3
	egg +COR+end	96.2	11.9	0.00	4	86.2
	egg +COR+egg*COR	96.2	11.9	0.00	4	86.2
	egg +COR+year*COR	97.0	12.7	0.00	4	85.0
	egg	98.7	14.4	0.00	2	92.7
	egg + year	99.4	15.1	0.00	3	91.4
	egg+ISL	100.1	15.8	0.00	3	92.1
	Null	101.5	17.2	0.00	1	97.5

Notes– *Wi  = * Akaike weight; *K* =  number of parameters; ISL  =  Incubation Shift Length, RS  =  Reproductive Success;

*Indicates interaction between variables; egg  =  egg volume index (Length × breadth^2^) for 1^st^ egg; pr_isl  =  duration of the previous incubation shift length ( =  partner's trip duration, days); end  =  date of incubation completion; X6H  =  wind speed 6 hours prior to colony arrival at 23:00; X72H  =  wind speed 72 h prior to colony arrival at 23:00; moon  =  proportion of moon (full moon: 14-day, new moon: 1-day); cloud  =  daily cloud opacity; COR  =  nest specific correlation coefficients; neg  =  cumulative days of egg neglect at each pair; ISL  =  mean incubation shift length at each nest; year  =  Year of observation.

### Effects of incubation shift lengths on reproductive success

The number of chicks (0, 1 or 2) that departed per pair (*n* = 41 in 2007, *n* = 35 in 2008) was best predicted by egg volume index, nest specific correlation coefficients (pair synchrony) and days of egg neglected. This model included nest ID as a random effect to account for individual heterogeneity in reproductive success. Reproductive success tended to increase with pair synchrony and egg size (1.68±0.65 chicks/pair in 2007; 1.23±0.94 chicks/pair in 2008). We also found support for interactions of differed between years and egg size, and mean incubation shift length. Egg size of non-deserting pairs was larger than that of deserting pairs (*n* = 133, 27 respectively, *t* = 2.18, *P* = 0.03), as previously found [42]. We also found support for interactions among variables of shift length and year in these best main-effect models.

### Foraging behavior

**Figure 3:**
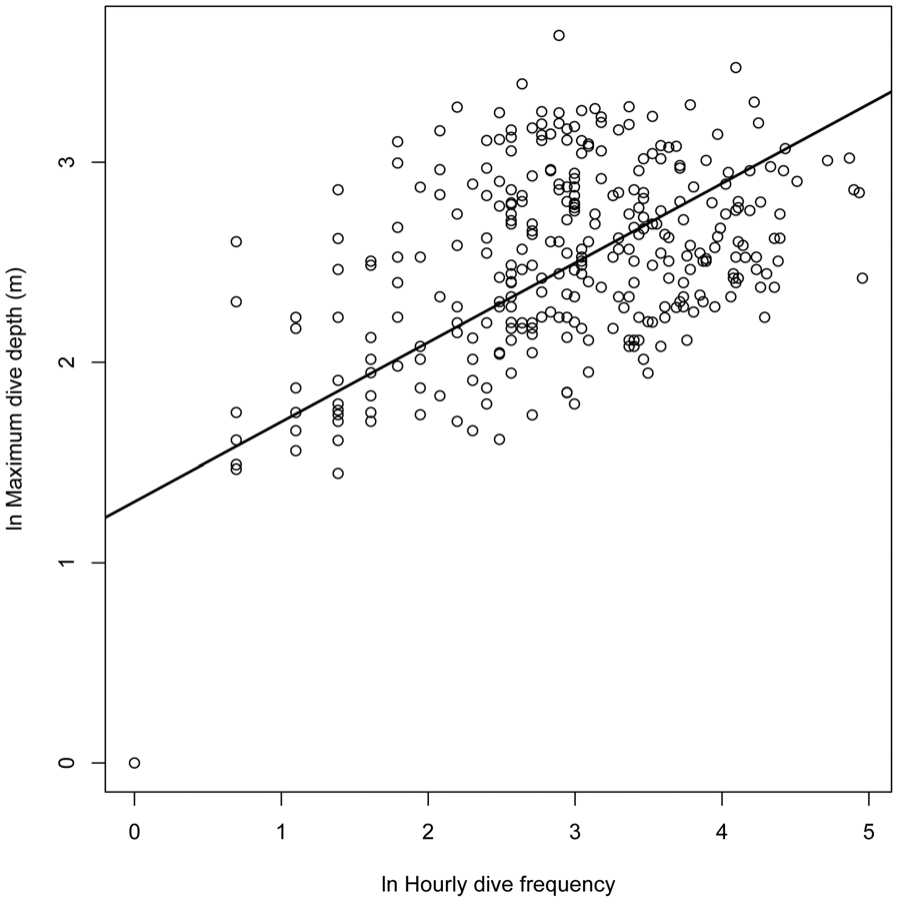
Relationship between natural logarithm (ln) hourly mean dive frequency and ln hourly maximum dive depth (*n* = 337).

**Figure 4:**
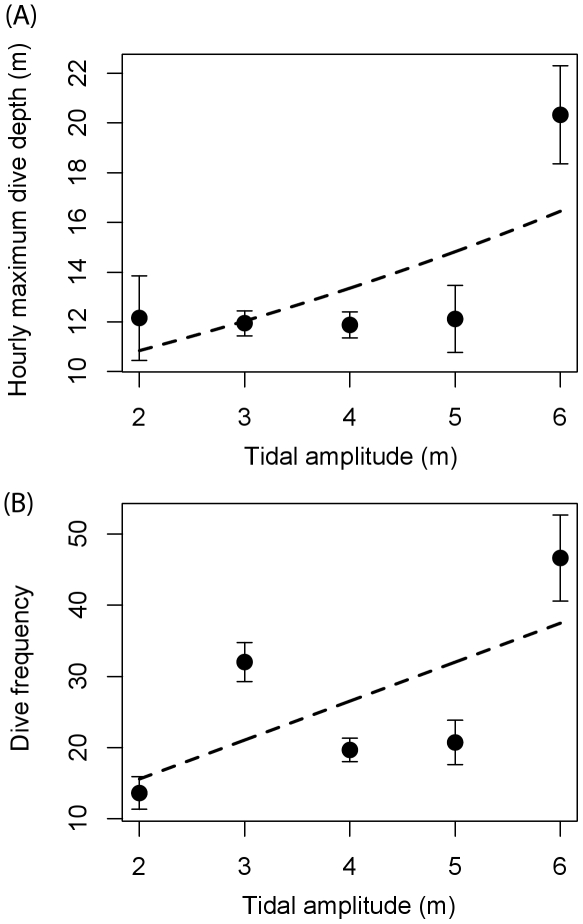
Diving behavior and tidal amplitude for ancient murrelets at Reef Island (*n* = 337). (A) Hourly maximum dive depth; (B) dive frequency increased with tidal amplitude. Data are presented as mean ± SE.

**Figure 5:**
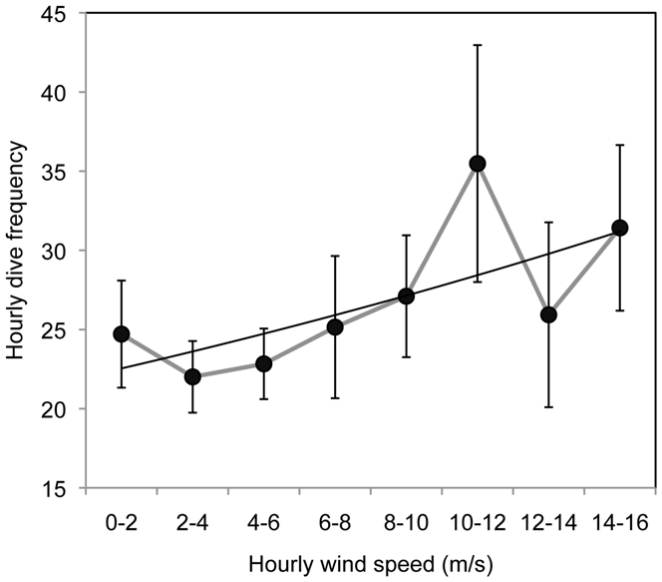
Hourly dive frequency increased with hourly mean wind speed (m/s) for ancient murelets at Reef Island (*n* = 337). Data are presented as mean ± SE.

**Figure 6:**
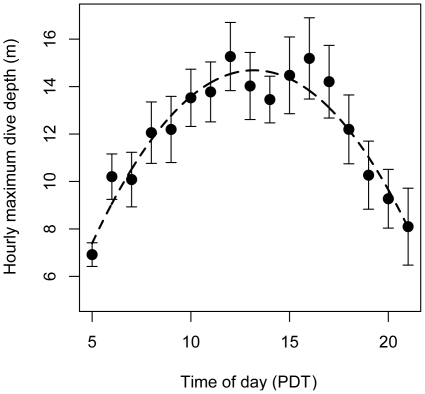
Distritbution of hourly maximum dive depth as function of time of day (*n* = 337). Data are presented as mean ± SE.

Little variation was found for hourly dive depth (mean  = 8.5 m, SE = 0.24, *n* = 327) and for dive frequency (mean  = 25.8, SE = 1.29, *n* = 327). The best model describing hourly maximum depth included wind speed, tide change and time of day (Table 3). Maximum dive depth per hour decreased with wind speed (Table 3). Larger tidal amplitude increased dive depth and dive frequency (Table 3, Fig. 3 and 4). Any model of maximum dive depth disregarding the effects of tide and time of day increased ΔAIC by an order of magnitude (from 7.4 to >70; Table 3), suggesting that both tide and time were important factors in this model. The best model of hourly dive frequency included wind speed, wave height, wind directions and tidal amplitude (ΔAIC  = 0.0, deviance  = 4623.3). Dive frequency increased with wind speed (Fig. 5), wave height and tidal amplitude (Table 3). Both dive depth and frequency were approximately constant outside of the periods of twilight or darkness (2100-0600), but declined rapidly to zero during the night, driving the strong relationship between those variables and time of day (Fig. 6) Dive frequency was slightly higher when wind was from the north.

**Table 3:** Results of AIC model comparison to explain hourly maximum dive depth and dive frequency of ancient murrelets.

Variables	Model	AIC	ΔAIC	*W_i_*	*K*	Deviance
Max dive depth	wind speed + wind direction (N-S)+ time of day + tide_change	581.4	0.0	0.33	5	569.4
(*n* = 337)	wind speed + time of day + tide_change	581.4	0.0	0.33	4	569.4
	wind speed + wind direction (N-S) + time of day	582.3	0.9	0.21	4	572.3
	wind speed + wind direction (N-S) + time of day + tide_change + tide difference	583.4	2.0	0.12	6	569.4
	time of day + tide_change	588.4	7.0	0.01	3	580.4
	time of day	588.8	7.4	0.01	2	582.8
	wind speed + wind direction (N-S)	653.4	72.0	0.00	3	645.4
	wind speed	654.2	72.8	0.00	2	684.2
	wind speed + wave height	654.7	73.3	0.00	3	646.7
	wind speed +wind direction (N-S) + wind direction (W∼E)	655.3	73.9	0.00	4	645.3
	tide_change	659.3	77.9	0.00	2	653.3
	Null	806.4	225.0	0.00	1	802.4
Dive frequency	wind speed + wave height + wind direction (N-S) + wind direction (W-E) + time of day + tide_change	4639	0.0	0.50	7	4623.3
(*n* = 337)	wind speed + wav height + wind direction (N-S) + wind direction (W-E) + time of day + tide_change + tide_difference	4639	0.0	0.50	8	4621.0
	wind speed + wave height + wind direction (N-S) + wind direction (W-E) + time of day	4658	19.0	0.00	6	4643.6
	time of day + tide_change + tide_difference	4722	83.0	0.00	4	4712.4
	time of day	4739	100.0	0.00	2	4732.7
	wind speed + wave height + wind direction (N-S) + wind direction (W-E)	4991	352.0	0.00	5	4978.6
	wind speed + wave height + wind direction (N-S)	5002	363.0	0.00	4	4991.7
	wind speed + wave height	5003	364.0	0.00	3	4995.1
	wind speed	5023	384.0	0.00	2	5016.5
	Null	5239	600.0	0.00	1	5234.7

**Notes–** *Wi  = * Akaike weight; *K*  =  number of parameters; Max dive depth  =  hourly maximum dive depth (m); Dive frequency  =  number of dives per hour; wind speed  =  hourly mean wind speed (km/h); wind direction (N-S)^a^  =  wind direction between North and South; wind direction (W-E)^ a^  =  wind direction between West and East; wave height  =  hourly wave height (m); time of day^ a^  =  time at daylight savings time in PST; tide change  =  height at next hour minus current tight; tide_difference  =  the highest tide minus lowest tide at a giving date ^a^circularly transformed.

## Discussion

To better understand the exceptionally long incubation shift length observed in *Synthliboramphus* compared to other auks [40], [43], [63], we conducted the first intensive study of incubation shift length taken over a period of two consecutive years. Incubation shift length varied among individual pairs of ancient murrelets, and shift duration was strongly correlated between pair members. The synchronization of an array of reproductive behaviors during breeding, including shift lengths, is critical for many seabirds, as previously reported [12], [46], [64].

We found significant variation in shift length among pairs, which is consistent with previous studies [40], [43]. Pairs members had a strong tendency to remain at sea for similar periods when off-duty, so that their shift lengths were strongly correlated. Our results suggest that foraging conditions and individual variations (*i.e*. parental quality), rather than predator avoidance, were the most important determinants of incubation shift length in ancient murrelets (Table 3). Variation in shift length among individual pairs could result from a variety of individual traits, including body condition and age. Body condition in particular is known to be a critical factor affecting incubation shift lengths in seabirds [12], [15], [19]. In this respect, long foraging trips usually increase energy intake for central-place foragers [29], [31], [32] and that could also apply to ancient murrelets [53]. However, long foraging trips require that partners have the physiological capacity to fast for an extended period of time, as the foraging time of one bird translates directly into fasting time for its partner. A pair that begins incubation with plentiful reserves should be capable of longer shifts than those starting with smaller reserves [65].

The incubation shift of one bird needs to be synchronized with the foraging shift of its mate so that the incubating bird does not depart before its mate returns, leaving the egg attended. This poor coordination of incubation shift length can increase the rate of breeding failure in seabirds [66]. There have been several proposals for explaining how pairs synchronize their behavior. Birds may tend to mate assortatively with respect to age and therefore experience. As a result, “high quality” (*i.e*., experienced) birds may choose high quality mates to avoid poor synchronization and reduce the need to compensate for a partner who requires more time at sea [66]. Although periods of uniform weather could cause successive incubation shifts to be similar in length, this cannot account for correlations in shift length over the entire incubation period, because there seems no reason why such synchronization would persist if weather changes.

In addition to the duration of the partner's previous shift, high wind speed was found to increase incubation shift length of ancient murrelets. This result is consistent with previous studies, which suggested that high wind speed led to poor foraging conditions at sea, and hence to longer shift lengths [16], [67], [68]. Dive frequency increased with wave height and wind speed, suggesting that birds need more foraging effort during rough seas that result from high wind speeds. Thus, ancient murrelets are likely to have lower foraging success and higher energy demands under unfavorable weather conditions, which leads to longer foraging trips and, from the partner's point of view, longer incubation shifts.

Tide may also affect foraging behavior [26], [68]. In some cases, tidal currents are strong enough to affect avian dive behavior by increasing travel costs [69], [70], [71]. In most cases, however, tide affects avian foraging behavior by altering the behavior or the abundance of prey [72]. Because plankton is susceptible to tidal currents, tide is expected to affect particularly the foraging of planktivorous species. Hence, it is not surprising that planktivores, including ancient murrelets, are often found in areas of tidal upwelling and are thought to follow the temporal progression of tidal changes [73]. Cassin's auklets changed diet and dove deeper during spring tides [26], while ancient murrelets selected the strongest tidal currents and fed more frequently during maximum tidal flow that at slack tides [74]. Because dive depth was the highest at around noon [Fig 6], it is likely that our birds followed the diel vertical migration of plankton to deeper depths during the middle of the day, as visibility should have been high throughout the time period examined [75].

On the other hand, ambient light affected nest arrival rate, and therefore egg neglect, but not incubation shift length, with older (brighter) moon decreasing nest arrival. Presumably, birds reduced nest visits under the threat of predation [76], [77]. This behavioral response to ambient light occurs in a number of nocturnal burrow-nesting seabirds, which are vulnerable on land [38], [39], [78], [79], [80]. Despite the effect of ambient light on nest arrival and egg neglect, light intensity did not affect incubation shift length. As the moon is at its brightest for only a few days every 28 days, the period of high predation risk is relatively short. Egg neglect is expected to occur due to a non-arriving partner and it is then likely that cloud opacity directly impacts predation at the colony. The influence of predation risk on incubation shift length depends on the length of these shifts; the threshold of light conditions that leads to increased predation risk is unknown and deserves further attention.

Overall, our results show that the foraging hypothesis and the predation avoidance hypothesis are not mutually exclusive. Seabirds have evolved a flexible approach to meet the requirements of their life-history strategy. If a bird decides not to return on a particular night due to predation risk, the on-duty bird may decide to leave the nest if its body reserves are critically depleted. However, these decisions are influenced by both foraging conditions at sea and by ambient light conditions. Reproductive success was related to incubation shift length, year, and egg size in ancient murrelets. Presumably, better quality or experienced birds [46] start their incubation shifts with higher reserves and consequently, are better able to accommodate variable foraging conditions [42]. Thus, better quality birds have higher reproductive success and longer shifts, which minimize predation risk. The idea that older birds have higher success is also supported by the relationship between egg size and desertion in this study (desertion probability inversely related to egg size), as older auks are known to lay larger eggs [81], [82].
